# Tracking the early depleting transmission dynamics of COVID-19 with a time-varying SIR model

**DOI:** 10.1038/s41598-020-78739-8

**Published:** 2020-12-10

**Authors:** Kian Boon Law, Kalaiarasu M. Peariasamy, Balvinder Singh Gill, Sarbhan Singh, Bala Murali Sundram, Kamesh Rajendran, Sarat Chandra Dass, Yi Lin Lee, Pik Pin Goh, Hishamshah Ibrahim, Noor Hisham Abdullah

**Affiliations:** 1 Institute for Clinical Research, Ministry of Health Malaysia, Setia Alam, Malaysia; 2 Institute for Medical Research, Ministry of Health Malaysia, Kuala Lumpur, Malaysia; 3 Heriot-Watt University Malaysia, Putrajaya, Malaysia; 4 Ministry of Health Malaysia, Putrajaya, Malaysia

**Keywords:** Infectious diseases, Epidemiology, Public health

## Abstract

The susceptible-infectious-removed (SIR) model offers the simplest framework to study transmission dynamics of COVID-19, however, it does not factor in its early depleting trend observed during a lockdown. We modified the SIR model to specifically simulate the early depleting transmission dynamics of COVID-19 to better predict its temporal trend in Malaysia. The classical SIR model was fitted to observed total (I total), active (I) and removed (R) cases of COVID-19 before lockdown to estimate the basic reproduction number. Next, the model was modified with a partial time-varying force of infection, given by a proportionally depleting transmission coefficient, $\beta_{t}$ and a fractional term, *z*. The modified SIR model was then fitted to observed data over 6 weeks during the lockdown. Model fitting and projection were validated using the mean absolute percent error (MAPE). The transmission dynamics of COVID-19 was interrupted immediately by the lockdown. The modified SIR model projected the depleting temporal trends with lowest MAPE for I total, followed by I, I daily and R. During lockdown, the dynamics of COVID-19 depleted at a rate of 4.7% each day with a decreased capacity of 40%. For 7-day and 14-day projections, the modified SIR model accurately predicted I total, I and R. The depleting transmission dynamics for COVID-19 during lockdown can be accurately captured by time-varying SIR model. Projection generated based on observed data is useful for future planning and control of COVID-19.

## Introduction

Compartmental mathematical models are critical for understanding the transmission dynamics of the Severe Acute Respiratory Syndrome Coronavirus 2 (SARS-CoV-2). These models are used to evaluate the impact of lockdown measures and various public health interventions during the COVID-19 pandemic^1–7^. The susceptible-infectious-removed (SIR) model is the simplest compartmental model used to describe the epidemic pattern of an infectious disease. It functions on the principle that individuals can be classified by their epidemiological status, based on their ability to host and transmit a pathogen. Most compartmental models assume that the number of cases increases exponentially until the epidemic can no longer be sustained due to the reduced number of susceptible individuals. This process continues until the number of infections drop, eventually leading to the extinction of an epidemic^8^.

In COVID-19 pandemic, the inadequacy of effective pharmaceutical remedies forced many countries to impose various public health interventions to flatten the epidemic curve. These measures included public lockdown, physical distancing, prohibition of gathering and schools closure to reduce the contact rate between individuals^9^. Other interventions such as contact tracing and quarantine were implemented to prevent the occurrence of transmission by isolating infected individuals before they could develop infectiousness^10^. However, the utility of any one intervention alone is likely to be limited, requiring multiple interventions to be combined to have a substantial impact on the dynamics of transmission^11^. Many countries also authorized legislative lockdown or movement control to optimize public response and compliance to those interventions.

In China, the epidemic growth of COVID-19 was successfully flattened within three months by strictly enforced movement restrictions and lockdown. The early extinction of COVID-19 was achieved with a high degree of compliance to the public health interventions^12^. In like manner, Malaysia first implemented a 3-week nationwide lockdown or Movement Control Order (MCO) beginning 18 March 2020. Thereafter, in response to the continuous growth of COVID-19 in the country, the MCO was extended twice until 12 May 2020. Malaysia further enforced the MCO for the fourth time until 9 June 2020; a total MCO duration of 12 weeks.

The aim of this study was to develop and validate a modified SIR compartmental model that factored in the early depleting transmission dynamics of COVID-19 and compare model predictions to observed COVID-19 cases during the lockdown period in Malaysia.

## Methods

### Model structure

**Figure 1:**
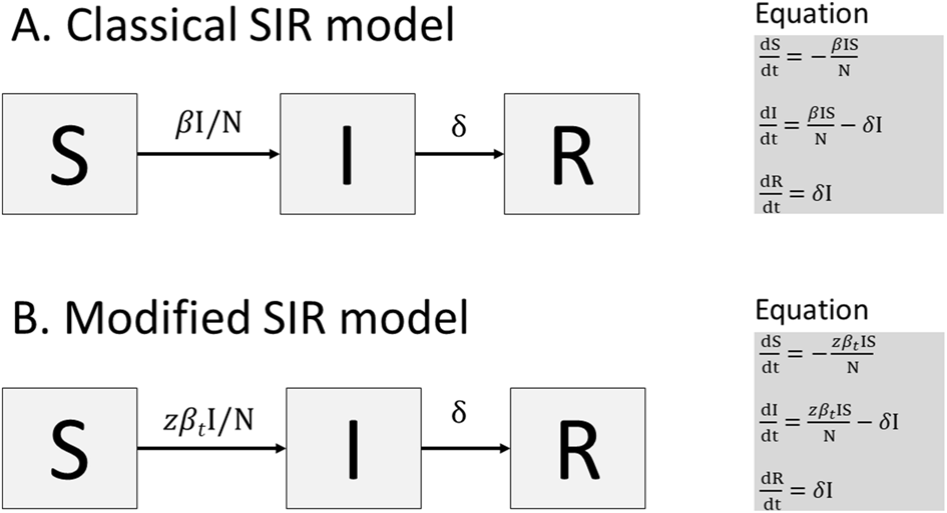
The compartmental structure of the classical SIR model and modified SIR model.

In countries affected by the COVID-19 pandemic, the SIR model provided the simplest framework that matched the reporting structure with the least underlying assumptions. Figure 1A shows the compartmental structure of a classical SIR model, with three state variables: S for susceptible, I for infectious and R for removed, and two transition rates: (1) Force of infection, $\beta {\text{I}}/{\text{N}}$ that controls the transition of individuals from S to I and (2) Removed rate, $\delta$ that controls the transition of individuals from I to R, respectively. The *δ* is the reciprocal of the infectious period and N is the sum of all three state variables. The force of infection is the rate at which individuals acquire an infection, which relies on the transmission coefficient, $\beta$ and the fraction of infectious individuals, ${\text{I}}/{\text{N}}$. The model assumes that the entire population remains equally susceptible during infection. As infected individuals were being isolated immediately once detected, we attributed the transition of individuals from compartment S to I to the transmission period and the transition of individuals from compartment I to R to isolation period or admission period.

The reproduction number, $\hat{R}$ is defined as the average number of secondary cases generated by an index case in a large entirely susceptible population. The basic reproduction number, $\hat{R}_{0}$ is estimated at the beginning of an outbreak when there is no population immunity or any deliberate intervention in disease transmission^13^. The $\hat{R}_{0}$ is a valuable concept to determine if an emerging infectious disease can spread in a population. When $\hat{R}_{0}$ > 1, an outbreak is expected to continue in the population. In this study, using the classical SIR model, the $\hat{R}_{0}$ was calculated by $\beta$ × transmission period whilst the effective reproduction number, $\hat{R}_{t}$ was estimated at the current state of the population.

Studies show that the $\hat{R}_{t}$ of COVID-19 gradually decreases over time, as a result of enforced lockdown and public health interventions^4,13,14^. To overcome the limitation of the classical SIR model in capturing the early reducing trend of COVID-19, a partial time-varying force of infection was incorporated into the SIR model. Figure 1B presents the modified SIR model with a partial time-varying force of infection, given by $z\beta_{t} I/N$, where $z\beta_{t}$ is the partial transmission coefficient at time *t*. The fractional term, $z$ allows the transmission dynamics to decrease with the number of infected individuals who can spread the coronavirus. A power decay log function representing gradually depleting $\beta_{t}$ over time $t$ is given by,

1 $$ \beta_{t} = \beta_{t = 0} (1 - p)^{t} , $$

where $p$ is the proportion of depletion between 0 and 1.

By incorporating the derivative of function (1) into ordinary differential equations (ODEs) as shown in Fig. 1B, the early depleting transmission dynamics of COVID-19 can be simulated.

**Figure 2:**
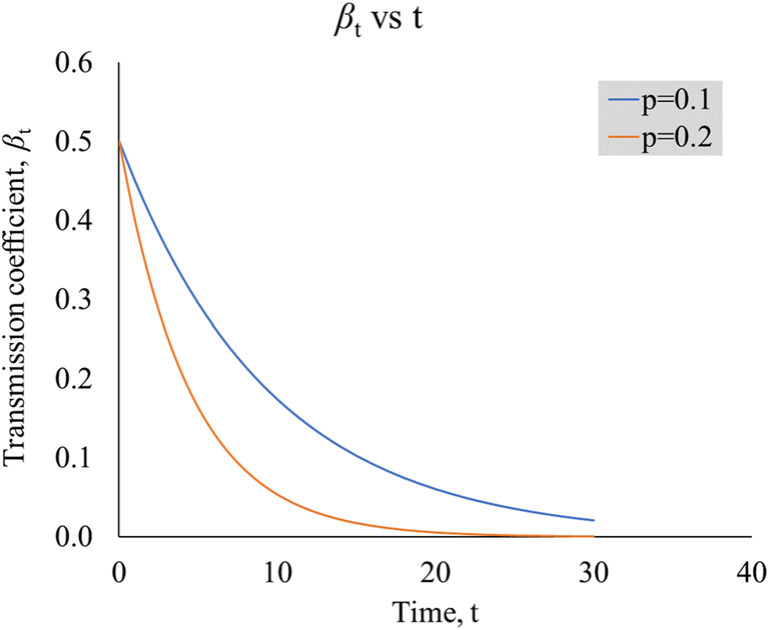
Graph of $\beta_{{\text{t}}}$ vs t, for two different values of p, illustrating the rate of depletion. The $\beta_{{{\text{t}} = 0}}$ is set at 0.5 (arbitrary).

The modified SIR model enables the outcome of lockdowns and public health interventions to be explicitly quantified by *p* and *z*. For instance, a larger value of $p$ signifies a more effective intervention in reducing contact rates and $\beta_{t}$ over time, whilst the value of z signifies the effectiveness of an intervention in preventing infected individuals from spreading the coronavirus. Figure 2 illustrates the observed $\beta_{t}$ at $p$ = 0.2 (20%), which is depleting faster than $p$ = 0.1 (10%). The $\hat{R}_{t}$ can be estimated by $z\beta_{t}$ × transmission period during the lockdown period.

### Data sources

The first wave of COVID-19 in Malaysia occurred between 25 January and 26 February 2020, involving only 22 individuals with 20 of them being oversea travelers. The second wave of COVID-19 emerged exponentially following a large religious gathering held in Sri Petaling, Kuala Lumpur between 27 February and 1 March 2020. The massive gathering involved more than 16,000 attendees, including many foreign nationals from countries with COVID-19 outbreak^15^. As of 26 April 2020, 2130 (37%) among 5780 confirmed cases were related to the Sri Petaling cluster^16,17^. The Ministry of Health (MOH) Malaysia reports on a daily basis the number of cumulative total cases, cumulative active cases, newly confirmed cases, recovered and death cases for COVID-19^18^. For this modeling, we denoted daily confirmed cases as I daily, cumulative total cases as I total, cumulative active cases as I and cumulative removed cases as R. Removed cases comprised of both recovered and death cases. The first day of Sri Petaling gathering (27 February 2020) was denoted as the start date of the second wave outbreak.

### The reporting structure of COVID-19 in Malaysia

All cases of COVID-19 were confirmed by real-time reverse transcriptase-polymerase chain reaction (RT-PCR) assays. Once confirmed, an infected individual was isolated immediately for treatment until recovery or death. Active cases were infected individuals who were still under treatment, whilst recovered cases were individuals who had been tested negative for COVID-19 by two RT-PCRs 24 hours apart. For this modeling, recovered individuals were assumed immune to re-infection.

**Figure 3:**
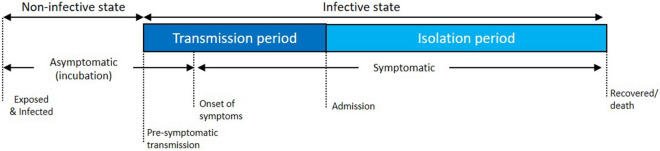
The phases of infection period for COVID-19.

Figure 3 shows various phases of the infection period for COVID-19. The infection period comprised of a non-infective and infective state and can be further divided into incubation, transmission and isolation period. The incubation period was the duration taken from being exposed to and infected by the coronavirus to the onset of symptoms. The incubation period for COVID-19 varies from 2 to 10 days on average^19^. The transmission period was the duration taken from the onset of symptoms until being isolated. Infected individuals are often asymptomatic and non-infective during the incubation period. However, pre-symptomatic transmission up to 3 days has been reported in several studies^20–22^. Hence, the transmission period might be longer, given the development of infectiousness before infected individuals could manifest symptoms^23^. The isolation period was the duration taken from isolation (admission) until recovery or death.

### Model fitting, projection and validation

Model fitting, projection and validation were performed in two stages. Firstly, the classical SIR model was fitted to observed cases of I and R between 27 February and 17 March 2020 to estimate the $\beta$ and $\hat{R}_{0}$ for COVID-19 in Malaysia. The compartment S took the initial value of 32.68 million, the population size of Malaysia in 2019^24^. The compartment ${\text{I}}$ and R took the initial values of 1 and 22 based on the observed number of cases reported on 27 February 2020.

Next, the modified SIR model was fitted to observed cases of I total, I and R in three sequences between 18 March and 28 April 2020 as follows:

- The first sequence involved observed cases from 18 March until 31 March 2020 (14 data points), with 7-day and 14-day projections, up to 14 April 2020.
- The second sequence involved observed cases from 18 March until 14 April 2020 (28 data points), with 7-day and 14-day projections, up to 28 April 2020.
- The third sequence involved observed cases from 18 March until 28 April 2020 (42 data points), with 7-day and 14-day projections, up to 12 May 2020.

The initial value for S remained. Then I total, I and R took the initial values of 790, 728 and 62, respectively, based on the observed number of cases reported on 18 March 2020. The $\beta_{t = 0}$ took the initial value estimated in the first stage.

Among all the state variables, I daily received the most attention from the public and was also the trickiest to predict. In this study, we tried to reproduce the temporal trend of I daily from the fitted I total with backward calculation. Comparison between projected cases and observed cases for I daily was performed using 5 days moving average (5 MA). As observed cases for I daily presented very high variation, high error in both model fitting and projection were expected.

The performance of the modified SIR model was evaluated using percent error (PE) and mean absolute percent error (MAPE). The MAPE which was given by $\frac{1}{n}\sum \left( {\frac{{\left| {{\text{Predicted}} - {\text{Observed}}} \right|}}{{{\text{Observed}}}}} \right) \times 100\%$, with a low value indicating high accuracy and vice versa. The PE is calculated by ($\frac{{{\text{Predicted}} - {\text{Observed}}}}{{{\text{Observed}}}}$) × 100%, with a positive value exhibiting overestimation and vice versa.

Model fitting was performed with least square and Markov Chain Monte Carlo methods, using the “shiny” modeling interface built and published by The Imperial College London (https://shiny.dide.imperial.ac.uk/infectiousdiseasemodels-2019/flu/). Built into the interface was an R-package called “odin” which operated a high-level language for describing and implementing ODEs. The actual solution of ODEs was processed with the “deSolve” package. Data was compiled and organized in Microsoft Excel 2019. Graphics were also produced using Microsoft Excel 2019.

### Ethics requirement

The study was registered with National Medical Research Register. No ethics approval was required.

## Results

Our modified SIR model outperformed the classical SIR model in tracking the infected population under lockdown circumstances. Some key features contributed to differences between the two models, regarding predictions of early disease depletion, transition of susceptible individuals to infected ones, the rate of infection spreading and effectiveness of model parameterization. Table 1 summarizes the differences between the modified and classical SIR models in capturing the early depleting trend of COVID-19 during the lockdown period.

**Table 1:** Differences between the modified and classical SIR model.

No	Modified SIR model	Classical SIR model
1	Early depletion of infection occurs due to strictly enforced lockdown and movement restrictions	Depletion of infection occurs due to continuously depleting susceptible until the epidemic can no longer be sustained
2	Transition of individuals from S to I is controlled by $z\beta_{t} {\text{I}}/{\text{N}}$. Transition of individuals from I to R is controlled by *δ*, where $\delta = \frac{1}{{\text{isolation period}}}$	Transition of individuals from S to I is controlled by $\beta {\text{I}}/{\text{N}}$. Transition of individuals from I to R is controlled by *δ*, where $\delta = \frac{1}{{\text{isolation period}}}$
3	The $\hat{R}_{t}$ is given by $z\beta_{t} \times {\text{transmission period}}$	The $\hat{R}_{0}$ is given by $\beta \times {\text{transmission period}}$
4	The transmission coefficient, $\beta_{t}$ is given by an exponential decay log function, $\beta_{t = 0} (1 - p)^{t}$, which allows $\beta_{t}$ to gradually decrease over time *t* with a fixed proportion of depletion, *p*	The transmission coefficient, $\beta$ is a fixed parameter
5	The transmission dynamics operate partially with fractional term, $z$	The transmission dynamics operate fully
6	Parameterization of *z*, *p* and $\delta$ can be done by fitting model to observed cases of COVID-19 during lockdown	Parameterization of $\beta$ and $\delta$ can be done by fitting model to observed cases of COVID-19 before lockdown

**Figure 4:**
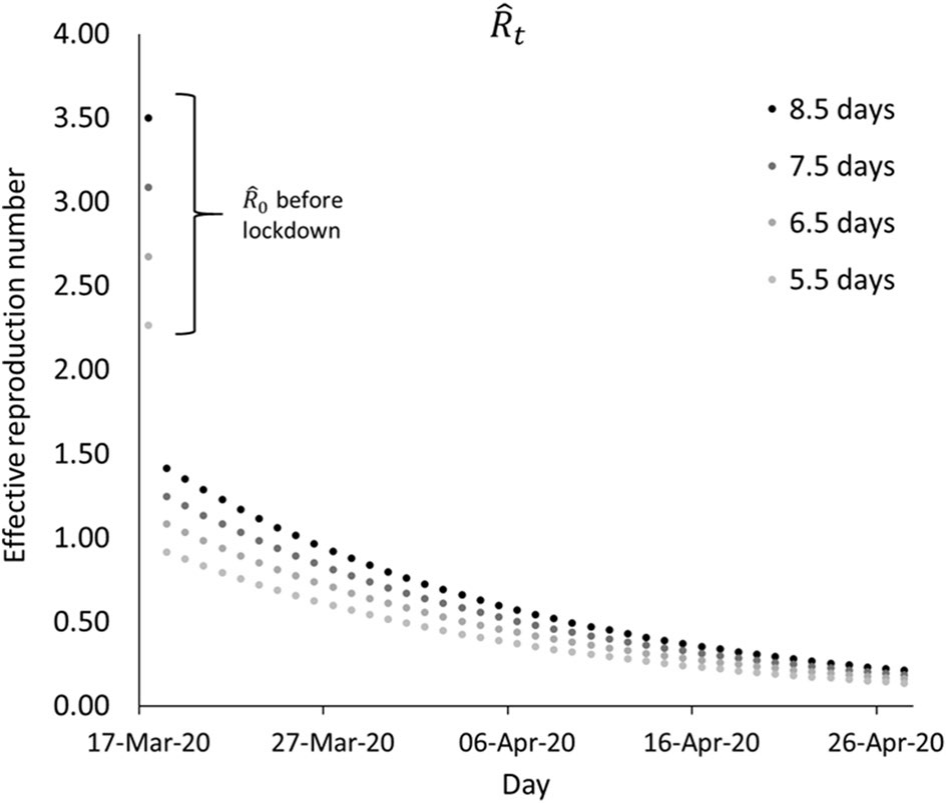
Estimated ${\hat{\text{R}}}_{0}$ prior to lockdown and ${\hat{\text{R}}}_{{\text{t}}}$ during lockdown.

The transmission, as effective reproduction number $\left( {\hat{R}_{t} } \right)$, decreased substantially after the lockdown (Fig. 4). Estimates of the classical SIR model placed $\hat{R}_{0}$ between 2.26 and 3.50, based on different transmission periods (5.5–8.5 days). On the other hand, the modified SIR model estimated the $\hat{R}_{t}$ to gradually decrease over time during the lockdown period towards values between 0.92 and 1.42, two to three times lower than the classical SIR model.

The transmission of coronavirus most likely occurred during a period from few days before onset of symptoms and admission or isolation as summarized in Table 2. The observed period from the onset of symptoms to admission among 86 patients was 5.5 days (95% CI 4.6–6.3 days). Hence, the transmission period could vary from 5.5 to 8.5 days, given that infected individuals could have developed infectiousness and transmitted the coronavirus up to 3 days prior to onset of symptoms^23^.

**Table 2:** Means of observed disease periods and 95% confidence interval (CI) for 156 COVID-19 patients.

Variables	Onset of symptoms to discharge, days		Onset of symptoms to admission, days		Admission to discharge, days	
	n	Mean [95% CI]	n	Mean [95% CI]	n	Mean [95% CI]
Overall	90	17.6 [16.4–18.8]	86	5.5 [4.6–6.3]	156	11.5 [10.6–12.3]
**Gender**						
Female	20	16.4 [14.3–18.5]	17	5.9 [3.9–7.9]	37	10.5 [8.9–12.2]
Male	70	18.0 [16.5–19.4]	69	5.4 [4.4–6.3]	119	11.8 [10.8–12.8]
**Status**						
Recovered	66	19.3 [18.1–20.6]	66	5.4 [4.3–6.4]	86	13.9 [13.2–14.6]
Death	24	12.9 [10.8–14.9]	20	5.9 [4.4–7.4]	70	8.5 [7.1–9.9]

*n *sample size, *CI *confidence interval.

Data source: Crisis Preparedness and Response Centre, MOH Malaysia (As of 14 April 2020).

### Transmission dynamics of COVID-19 before lockdown

**Figure 5:**
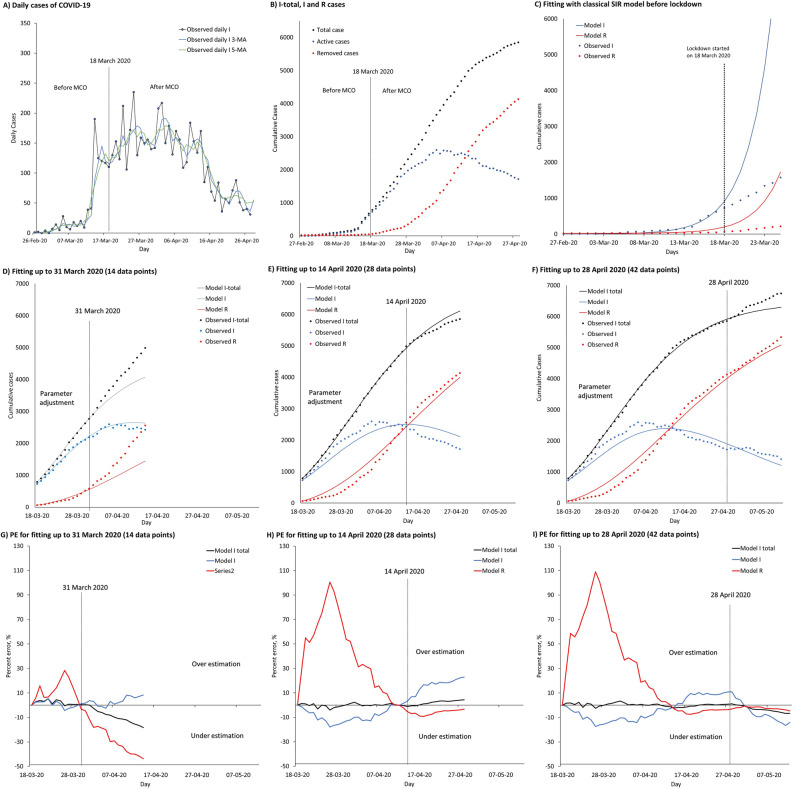
Transmission dynamics of COVID-19 in Malaysia before and during lockdown, fitted with classical and modified SIR models. Parts **(A)** to **(I)** represent observed daily and cumulative cases of COVID-19 cases in Malaysia, fitted classical SIR and modified models, projections and percent errors (PE) over time. (**A**) Observed I daily cases, smoothened with 3 MA and 5 MA before and after movement control order (MCO). (**B**) Observed I total, I and R cases over time before and after the MCO. (**C**) Fitted classical SIR model to observed cases of I and R from 27 February to 17 March 2020 before lockdown. (**D**) Fitting and projection up to 14 April 2020. (**E**) Fitting and projection up to 28 April 2020. (**F**) Fitting and projection up to 12 May 2020. (**G**) PE of fitting and projection up to 14 April 2020. (**H**) PE of fitting and projection up to 28 April 2020. (**I**) PE of fitting and projection up to 12 May 2020.

The growth rate on the number of infected cases fell immediately after the lockdown (Fig. 5). Before lockdown, a sudden surge in I daily caused an exponential increase observed in both I total and I, which warranted the government to implement a lockdown in the country. Daily moving averages on infected population showed similar results for 3 days and 5 days (Fig. 5A). With Movement Control Order (MCO), the epidemic curve of I daily was flattened before end of March 2020. A week later, the epidemic curve of I was flattened as the R started to surpass the decreasing I daily, as shown in Fig. 5B.

The classical SIR model was successfully fitted to both observed cases of I and R between 27 February and 17 March 2020, with an estimated *β* of 0.4114 (Fig. 5C). The fitted model showed that the initial dynamics of transmission mainly depended on the high infection rate, and not much affected by the low removing rate. The difference between the projected cases and observed cases for I uncovered an early reducing trend in COVID-19 during the MCO, which could no longer be predicted by the classical SIR model. The estimated $\hat{R}_{0}$ values were in line with values reported in other countries, such as South Korea and Italy, through mathematical modelling. The $\hat{R}_{0}$ for South Korea was estimated at 2.6 (95% CI 2.3–2.9) and 3.2 (95% CI 2.9–3.5) with transmission starting dates on 31 January and 5 February 2020. The $\hat{R}_{0}$ for Italy was estimated at 2.6 (95% CI 2.3–2.9) and 3.3 (95% CI 3.0–3.6) with the transmission starting dates on 5 February and 10 February 2020^5^.

### Early depleting transmission dynamics of COVID-19 during the lockdown

The modified SIR model adjusted well to observed cases for I total, I and R in all three sequences, and reproduced the correct temporal patterns for all compartments, especially with 28 and 42 data points (Fig. 5D–F). This showed that the modified SIR model had overcome the limitation of the classical SIR model in predicting the early depleting trend of COVID-19 under a public lockdown situation.

Overestimation was found in R in all three sequences during fitting, leading to underestimated R in projection. The overestimation in R during fitting did not affect the accuracy in projection. Nonetheless, underestimation was found in I during fitting, leading to overestimated I in projection. Graphs of percent error (PE) over time showed consistently reliable fitting and projection, in particular sequences with 28 and 42 data points (Fig. 5G–I).

The highest model accuracy expressed as lowest mean average percent error (MAPE) was achieved for I total (MAPE 0.97–2.13%), followed by I (MAPE 2.25–8.85%), I daily (MAPE 4.86–9.78%) and R (MAPE 12.30–40.61%), respectively. In both 7-day and 14-day projections, the lowest MAPE was achieved for I total (MAPE 1.24–5.58%), followed by I (MAPE 2.16–13.62%), R (MAPE 1.52–20.6%) and I daily (MAPE 30.99–50.29%), respectively. The projection accuracy improved slightly without imported cases (ICs), as shown in Table 3.

**Table 3:** Summary of estimated values for parameters p, z and δ in all three sequences and MAPE for model fitting, 7-day and 14-day projections, with inclusion and exclusion of imported cases (ICs).

Periods State variables	Data points	Parameter estimates			Fitting MAPE (%)		7-day projection MAPE (%)		14-day projection MAPE (%)	
		*z*	*p*	*δ*	Mean	(Min–max)	Mean	(Min–max)	Mean	(Min–max)
**18 Mar–31 Mar 2020**	14	0.4374	0.0784	0.025						
Including ICs										
I total					2.13	(0.31–5.35)	5.58	(0.09–9.67)	9.8	(0.09–18.33)
I					2.25	(0.03–5.19)	2.16	(0.76–3.71)	4.14	(0.76–8.38)
I daily–5 MA					4.86	(0.22–11.62)	30.99	(21.96–36.17)	38.05	(21.96–57.35)
R					12.30	(3.28–28.44)	20.6	(11.58–29.66)	29.5	(11.58–43.64)
Excluding ICs										
I total					–	–	4.05	(0.60–7.33)	7.7	(0.60–15.50)
I					–	–	3.53	(0.44–6.94)	8.1	(0.44–16.38)
I daily–5 MA					–	–	25.38	(16.42–31.60)	33.08	(16.42–54.94)
**18 Mar**–**14 April 2020**	28	0.3914	0.0450	0.042						
Including ICs										
I total					0.97	(0.02–4.08)	1.24	(0.19–2.78)	2.42	(0.19–4.42)
I					8.68	(0.27–17.96)	13.62	(6.84–18.56)	16.86	(6.84–22.87)
I daily–5 MA					5.54	(0.16–16.06)	35.79	(3.03–61.74)	31.69	(3.03–61.74)
R					40.61	(0.02–100.67)	7.62	(5.74–9.21)	6.01	(3.26–9.21)
Excluding ICs										
I total					–	–	1.91	(0.36–4.56)	3.96	(0.36–7.22)
I					–	–	15.79	(6.98–23.08)	22.52	(6.98–34.88)
I daily–5 MA					–	–	71.28	(7.57–136.71)	66.70	(7.57–136.71)
**18 Mar**–**28 April 2020**	42	0.4047	0.0466	0.050						
Including ICs										
I total					1.07	(0–3.43)	1.85	(0.11–3.57)	3.58	(0.11–6.65)
I					8.85	(0.96–17.36)	5.51	(0.22–9.38)	8.78	(0.22–16.45)
I daily–5 MA					9.78	(0.26–42.70)	50.29	(25.54–58.64)	53.96	(25.54–67.06)
R					31.50	(0.54–108.92)	1.52	(0.80–2.09)	2.40	(0.80–4.67)
Excluding ICs										
I total					–	–	0.90	(0.18–1.53)	1.71	(0.18–3.81)
I					–	–	5.23	(0.97–10.62)	3.79	(0.22–10.62)
I daily–5 MA					–	–	35.10	(11.03–48.37)	44.44	(11.03–64.71)

*MAPE *mean absolute percent error, *ICs *imported cases, *I total *cumulative total cases, *I *cumulative active cases, *I daily *daily confirmed cases, *R *cumulative removed cases (recovered + death), *min *minimum, *max *maximum, *MA *moving average, *z *fractional term, *p *proportion of depletion, *δ *removed rate.

**Figure 6:**
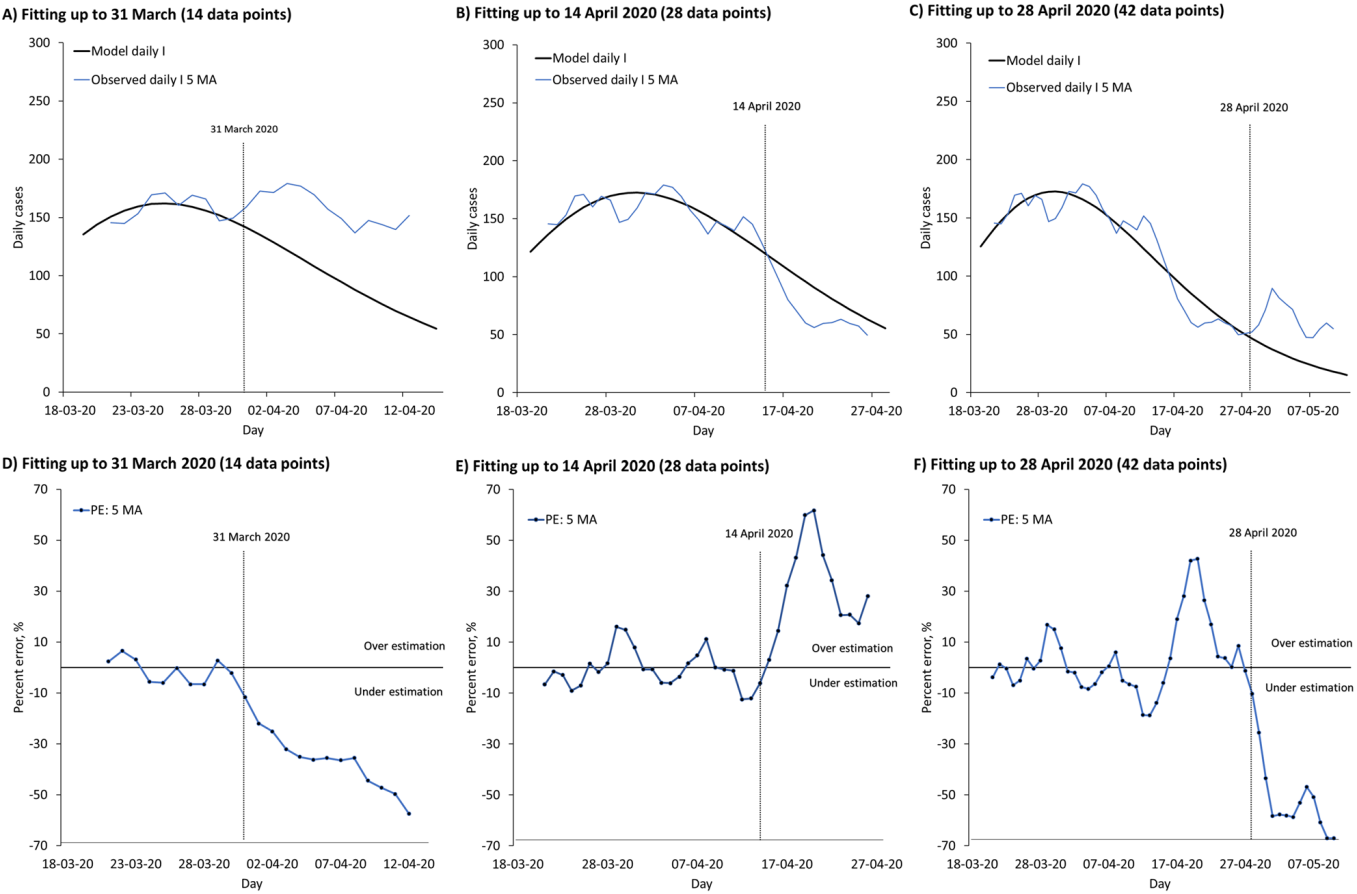
Temporal trend of daily confirmed COVID-19 cases in Malaysia. Part **(A)** to **(F)** present temporal trend of daily I for COVID-19 and associated percent error (PE) over time in Malaysia. (**A**) Fitting up to 31 March 2020, with projection up to 14 April 2020. (**B**) Fitting up to 14 April 2020, with projection up to 28 April 2020. (**C**) Fitting up to 28 April 2020, with projection up to 12 May 2020. (**D**) PE of fitted model from 18 to 31 March 2020, with projection up to 14 April 2020. (**E**) PE of fitted model from 18 March to 14 April 2020, with projection up to 28 April 2020. (**F**) PE of fitted model from 18 March to 28 April 2020, with projection up to 12 May 2020.

**Figure 7:**
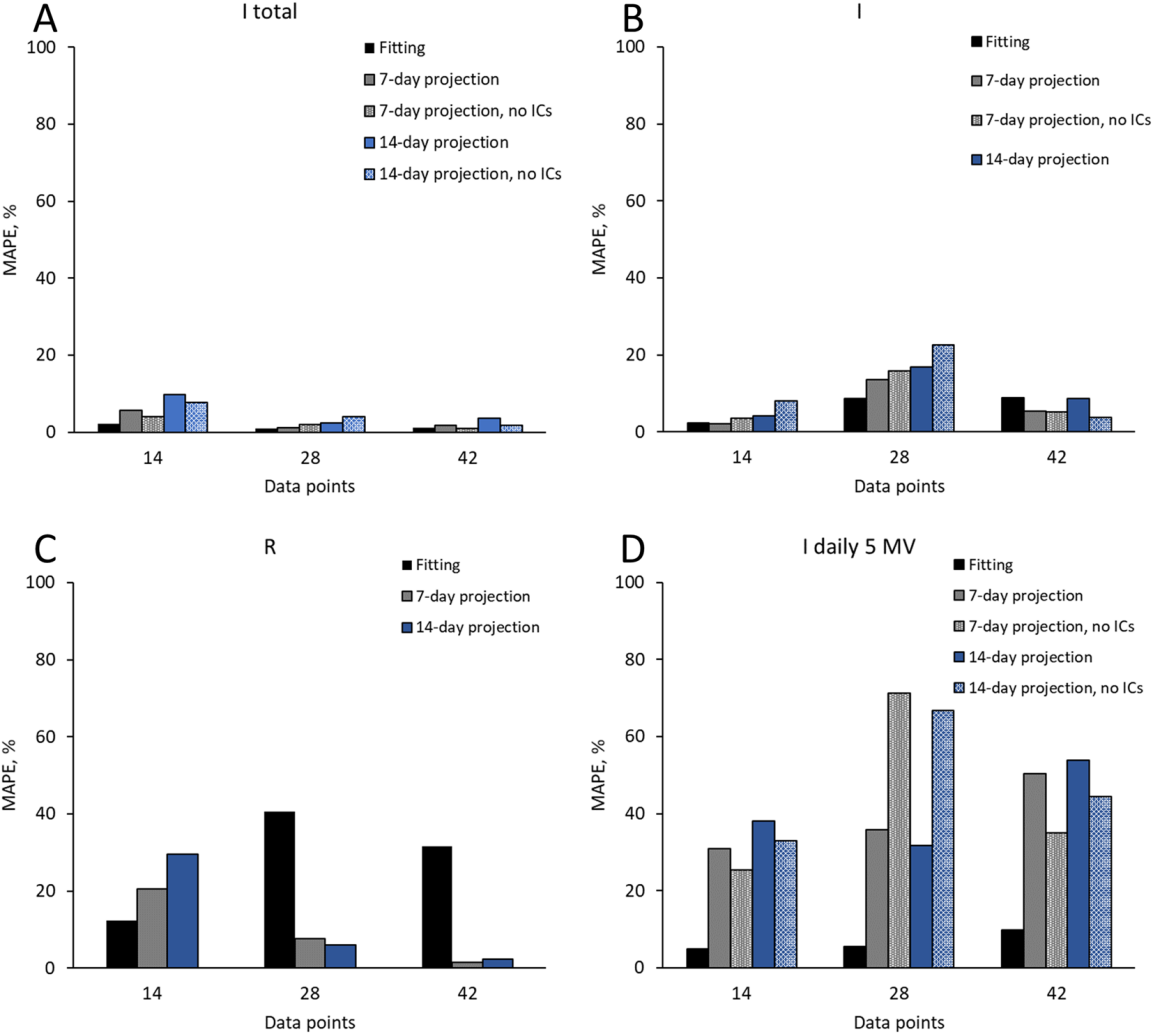
The impact of data points on MAPE of model fitting and projection. Part (**A**) to (**D**) present MAPE of model fitting and projection with 14, 28 and 42 data points for I total, I, R and I daily, with and without imported cases (ICs).

The modified SIR model successfully captured the I daily temporal trend (Fig. 6A–C). Due to the high variation, high MAPE was found in projection even with comparatively low MAPE in model fitting (Fig. 6D–F). Overall, the projection improved when fitted on more data points for I total, I and R (Fig. 7).

## Discussion

In general, compartmental models assume that the epidemic growth of an infectious disease is limited by the proportion of susceptible individuals^8^, and therefore may fail to predict the early depleting trend observed in COVID-19. Instead, the modified SIR model factors in changes in host behaviour and interaction, such as reduced contact rate, and can provide a better prediction for COVID-19 especially under a public lockdown situation with high degree of compliance as observed in Malaysia. The objective of predicting the early depleting transmission dynamics of COVID-19 is achieved by using a time-varying exponential decay log function for $\beta_{t}$ with a fractional term, z. This signifies the importance of incorporating valid principles in modeling for COVID-19.

To match the reporting structure of COVID-19 in Malaysia, we maintained the three compartmental structure and minimal underlying model assumptions. This increased the usability of the modeling findings, especially for stakeholders with limited understanding about mathematical models. The predictions of the modified model were used by the Ministry of Health Malaysia periodically to assess the requirement to extend the movement control order (MCO) and balance with opening of economic sectors during the MCO. The temporal trend of daily confirmed cases was useful to inform the public on the importance of maintaining physical distancing and good personal hygiene to prevent the spread of COVID-19^18^.

As of 11 May 2020, the total number of confirmed COVID-19 cases in Malaysia reached 6726, with 3821 (56.8%) cases being patients under investigation and their close contacts, 2345 (34.8%) cases from the Sri Petaling cluster, 353 (5.2%) cases from quarantine center (imported cases) and 207 (3.1%) cases from active surveillance^18^. The data showed that public health interventions such as contact tracing and quarantine measures effectively detected and isolated a significant proportion of infected individuals before they could spread the virus to others. This justified the use of a fractional term to adjust the overall transmission dynamics of COVID-19 during the lockdown period.

The difference between the estimated $\hat{R}_{0}$ and initial values of $\hat{R}_{t}$ signifies a prompt interruption or breaking in the transmission dynamics of COVID-19, a consequence of lockdown and movement restriction. Although nationwide lockdown and movement restriction are associated with a huge socio-economic burden, the observed instant decline in the transmission dynamics of COVID-19 give credence to the need for such drastic intervention to break the spread of COVID-19.

At first, the modified SIR model approximates the transmission dynamics of COVID-19 depleting at a higher rate of 7.8% (*p* = 0.078) per day but then decelerating to 4.7% (*p* = 0.047) per day eventually. The change in the proportion of depletion might be related to reduced compliance, especially in physical distancing during the lockdown. Also, the model shows that the transmission dynamics of COVID-19 might occur at a decreased capacity of about 40% (*z*: 0.3914–0.4313) during the lockdown period. The high MAPE in model fitting for compartment R could be caused by the use of a fixed parameter *δ* for the transition of individuals from compartment I to R, which is rigid in describing the inconsistent removed rate or isolation period over time. Nevertheless, the modified SIR model performed well in projecting the number of R cases over time with increased data points.

The high MAPE in projection for I daily is expected because of the underlying variation of daily confirmed cases. Projection of I daily is crucial as daily confirmed cases receive more attention from the public and stakeholders than other state variables. Besides, it is also a more sensitive indicator of a rebound in transmission and public adherence to control measures. The projected temporal trends are useful in decision making for extending or lifting of lockdown.

Our study highlights a few valuable features of the modified SIR model in capturing the depleting trend of COVID-19 as a result of lockdown and combined public health interventions. The model also produces realistic projection based on observed data, especially for I total, I and R state variables. The modified SIR model quantifies the transmission dynamics of COVID-19 with a fixed proportion of depletion per day, and in addition provides an intuitive parameter for comparing the impact of different public health strategies. Initial forecasts of the modified SIR model were informative, but less precise as the projections had limited data points. However, over time, the accuracy of the modified SIR model improved significantly with more data points being available for curve adjusting.

There are several limitations to the modified model. First, the modified SIR model is as good as the observed data captured and reported, not beyond. Its performance can be affected by reporting issues such as change of reporting structure and definition, testing backlog, inconsistent screening rate or yield and others. Second, projection based on observed data is valid only if the past conditions are followed without substantial changes. Refitting using most recent cases and time points might be appropriate if substantial changes are found.

## Conclusion

Our results show that lockdown and movement restriction are effective. They serve as the prerequisite to the early depletion of COVID-19, which can be accurately predicted by the SIR model modified with a partial time-varying force of infection. The time-varying SIR model helps to quantify the impact and effectiveness of lockdown and public health measures against the COVID-19, and are useful in the planning for the control of COVID-19 pandemic.
